# Acute Kidney Injury (AKI) in Young Synthetic Cannabinoids Abusers

**DOI:** 10.3390/biomedicines10081936

**Published:** 2022-08-10

**Authors:** Stefano D’Errico, Martina Zanon, Davide Radaelli, Monica Concato, Martina Padovano, Matteo Scopetti, Paola Frati, Vittorio Fineschi

**Affiliations:** 1 Department of Medicine, Surgery and Health, University of Trieste, 34137 Trieste, Italy; 2 Department of Anatomical, Histological, Forensic and Orthopedic Sciences, Sapienza University of Rome, 00161 Rome, Italy; 3 Department of Medical Surgical Sciences and Translational Medicine, Sapienza University of Rome, 00189 Rome, Italy

**Keywords:** synthetic cannabinoids, intoxication, acute kidney injury, acute tubular damage

## Abstract

**Background**. Synthetic cannabinoid-related acute kidney injury represents an increasingly important public health issue due to the diagnostic challenges given by low clinical suspicion of the disease and the frequent undetectability in routine drug tests. **Methods**. A systematic literature search on PubMed was carried out until 31 January 2022. Case reports, case series, retrospective and prospective studies, as well as reviews on acute kidney injury related to the consumption of synthetic cannabinoid were searched. **Results**. The systematic review process selected 21 studies for a total of 55 subjects with synthetic cannabinoid-induced acute kidney injury. Renal damage was demonstrated by elevated serum creatinine levels in 49 patients (89%). On renal ultrasound, the most frequent finding was an increase in cortical echogenicity. Renal biopsy, performed in 33% of cases, revealed acute tubular damage, acute tubulointerstitial nephritis, and acute interstitial nephritis, in decreasing order of frequency. **Conclusion**. Prompt identification and treatment of synthetic cannabinoid-related acute kidney injury represent a sensitive public health goal both for the acute management of damage from synthetic cannabinoids and for the prevention of chronic kidney disease.

## 1. Introduction

Synthetic cannabinoids (SCs) are marijuana-like, non-natural compounds, synthesized in the early 1960s [1,2]. Despite having a molecular structure similar to Δ9-tetrahydrocannabinol (THC), the main active compound in cannabis, these molecules have different pharmacodynamic and pharmacokinetic characteristics with greater potency. Since the 2000s, molecular variations of SCs have begun to appear and be commercialized due to low costs and undetectability in routine drug tests. To date, SCs can be found in herbal mixtures and bath salts. SCs can be smoked, ingested, or insufflated and have psychoactive effects due to the full agonist action on type 1 (CB1) and type 2 (CB2) cannabinoid receptors. Regarding localization, CB1 receptors are in the central nervous system and are associated with the psychotropic effects; CB2 receptors are in immune and hematopoietic cells where they mediate a negative immunomodulatory effect [3,4]. It appears that both receptors are expressed in the kidneys, although their role remains poorly understood [5]; some studies report that these receptors may be involved in renal blood flow regulation and sodium reabsorption. The higher receptor affinity of SC, compared to THC, results in stronger adverse effects such as agitation, aggressive behavior, drowsiness, coma, panic attacks, psychosis, hallucinations, delirium, confusion, convulsions, dizziness, mydriasis, nausea, vomiting, chest pain, tachycardia, rhabdomyolysis, liver damage, acute myocardial infarction, respiratory depression, and acute kidney injury [6]. According to literature, the prototypical presentation of the SCs user is a young (age 13–40) male with tachycardia, agitation, nausea, and vomiting.

Renal problems due to SCs account for less than 1% of all cases of intoxication, and acute kidney injury (AKI) is the most common pathology [7,8,9,10,11,12]. Since 2004, the definition of AKI replaces that of acute renal failure and represents an abrupt decrease in kidney function, which encompasses both injury and impairment [13]. Diagnosis is based on an acute decrease in glomerular filtration rate associated with increased serum creatinine (sCr) values. Diagnostic criteria include absolute increase in sCr—at least 0.3 mg/dL within 48 h or 50% increase in sCr from baseline within 7 days—or a urine volume of less than 0.5 mL/kg/h for at least 6 h [14]. Acute kidney injury rarely has a unique and distinct pathophysiology. The classification of AKI includes several pre-renal, post-renal and intrinsic causes. The pre-renal and post-renal forms are a consequence of extrarenal diseases such as hypovolemia, hypoperfusion or obstruction of the urinary tract; the persistence of the causal conditions determines the evolution towards kidney damage. Concretely, SCs are known for their negative inotropic effects, which can cause a decrease in renal blood flow; such an effect can be exacerbated in the presence of cannabinoid hyperemesis syndrome, typical for long-term users and characterized by nausea, periodic vomiting, abdominal pain, or discomfort [15]. Intrinsic causes of AKI are related to tubular, glomerular, interstitial, or vascular damage. The most common form of intrinsic AKI is acute tubular necrosis (ATN), followed by interstitial damage due to acute interstitial nephritis (AIN) and glomerular damage due to acute glomerulonephritis [16]. In the case of synthetic cannabinoid use, ATN is the leading cause of AKI and it could be related to rhabdomyolysis or directly drug-induced. Drug-induced AKI could be due to the direct toxic effects in tubular cells of the drug itself, the metabolites, or the other substances added to the mixtures. The role of tubular cells is to secrete and absorb filtrated molecules, as well as concentrate urine; such a function obviously exposes tubular cells to high concentrations of toxic molecules. Unfortunately, it is still not clear which components are responsible of ATN [17]. To date, the most relevant theory about AKI due to SC toxicity is a multifactorial mechanism; in many cases, ATN and AIN are due to volume depletion in association with the toxic effects of SCs or other compounds added into the mixtures. Treatment for AKI includes observation and supportive care through intravenous fluids for volume expansion; in severe cases, hemodialysis can be used. In cases of acute interstitial nephritis, steroids may be indicated. To date, there is no antidote for SC intoxication [18,19]. 

The present systematic review, according to PRISMA 2020 guidelines (registration code 340207), aims to summarize the available evidence regarding kidney damage induced by synthetic cannabinoids; the secondary objective is the preparation of an orientation tool for the professionals involved with different roles in the management of the problem.

## 2. Materials and Methods

### 2.1. Search Criteria and Critical Appraisal

**Figure 1:**
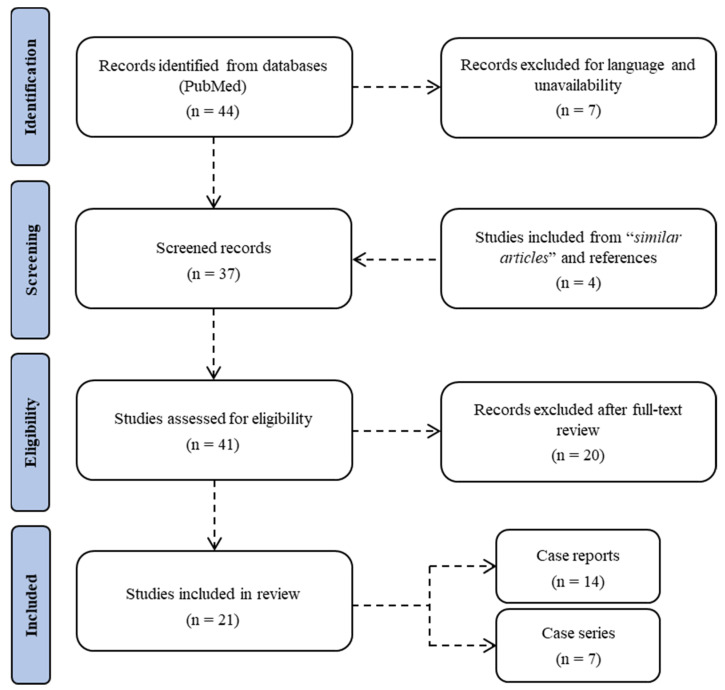
Flow chart of the systematic literature search according to PRISMA 2020 guidelines.

A systematic literature search on PubMed was carried out until 31 January 2022 (Figure 1). No time restrictions were applied. The review was conducted using MeSH terms, Boolean operators, and free-text terms to broaden the research. Studies focusing on acute kidney injury related to the consumption of synthetic cannabinoids were initially searched using the terms “((AKI) OR (acute renal injury)) AND ((SCs) OR (synthetic cannabinoids))” in title, abstract, and keywords. Study design included case reports, case series, retrospective and prospective studies, as well as reviews. No unpublished or gray literature was searched. A total of 44 articles were found in the database. Of these, 5 were excluded because they were not published in the English language and 2 were left out due to unavailability. The evaluation of references during full-text screening allowed the inclusion of a further 4 studies. After evaluation of abstracts and full texts, 20 articles were excluded because they were deemed not relevant to the research topic. Data from each included study were extracted using Microsoft Excel spreadsheets, including information on authors, publishing years, nation, sample size, gender, age, synthetic cannabinoid, other drugs, symptoms, hospitalization time, kidney imaging and biopsy, laboratory results, hemodialysis, and mortality.

### 2.2. Causality Assessment

The enrolled cases were subjected to a causality assessment using the Naranjo Adverse Drug Reaction Probability Scale to corroborate the findings of the review [20]. 

The algorithm, developed for use in controlled studies or the registration of new drugs, was chosen to confer scientific objectivity to the evaluation of the role of synthetic cannabinoids in the genesis of acute kidney injury. Briefly, the implementation of the scoring system implied the evaluation of each case for:

- Existence of previous adverse reaction reports;
- Temporal correlation between the substance intake and the adverse event;
- Regression upon withdrawal of the substance or after administration of a specific antagonist;
- Appearance of the adverse reaction to a new administration of the substance;
- Existence of alternative causes underlying the adverse reaction;
- Appearance of the adverse reaction to the placebo administration;
- Detection of the substance in biological samples with a toxic dosage;
- Severity of the adverse reaction directly proportional to the dose;
- Presentation of the adverse reaction following the intake of the same or similar substance;
- Objective confirmation of the existence of the adverse event.

The result consists of a numerical value included in a range from −4 to +12; the score is directly proportional to the probability of the existence of an adverse drug reaction and allows stratification into 4 classes: definite (≥9), probable (5 to 8), possible (2 to 4), and doubtful ADR (<2).

## 3. Results

A total of 21 studies, 7 case series and 14 case reports, were included in the review (Table 1); 17 reports came from the USA, 2 from Turkey, 1 from Belgium, and 1 from Southwest Asia.

**Table 1:** Features of the primary studies included.

Authors and Year of Publication	Country	Total Cases	AKI Cases	Gender (M:F)	Age Range	SC	Drug Association
Argamany et al. (2015) [21]	USA	1	1	1 (M)	27	Unknown	No
Armstrong et al. (2019) [22]	USA	6	5	4:1	17–47	Known (2) Unknown (3)	Yes (1) No (4)
Behonick et al. (2014) [23]	USA	4	1	4 (M)	27	Known	Yes
Bhanushali et al. (2013) [24]	USA	4	4	4 (M)	20–30	Known (4)	No (4)
Buser at al. (2014) [25]	USA	9	9	9 (M)	15–27	Known (1) Unknown (8)	No (9)
Cooks et al. (2016) [26]	USA	1	1	1 (M)	17	Unknown	Yes
Ergül et al. (2015) [27]	Turkey	6	3	3 (M)	21–27	Known (3)	Yes (2) No (1)
Gudsoorkar et al. (2015) [28]	USA	1	1	1 (M)	26	Unknown	No
Kamel et al. (2015) [29]	USA	1	1	1 (M)	65	Unknown	No
Karass et al. (2017) [30]	USA	1	1	1 (M)	20	Unknown	Yes
Kazory et al. (2013) [31]	USA	1	1	1 (M)	22	Unknown	No
CDC (2013) [32]	USA	16	16	15:1	15–33	Known (11) Unknown (5)	No (16)
Sherpa et al. (2015) [33]	USA	1	1	1 (M)	42	Known	No
Sinangil et al. (2016) [34]	Turkey	1	1	1 (M)	42	Known	No
Srisung et al. (2015) [35]	USA	3	3	3 (M)	31–32	Unknown	No (3)
Stuart et al. (2020) [36]	Southwest Asia	1	1	1 (M)	32	Known	No
Thornton et al. (2013) [37]	USA	1	1	1 (M)	26	Known	No
Ukaigwe et al. (2014) [38]	USA	1	1	1 (M)	28	Known	No
Van Rafelghem et al. (2021) [39]	Belgium	1	1	1 (M)	22	Known	Yes
Zarifi et al. (2017) [40]	USA	1	1	1 (M)	21	Known	No
Zhao et al. (2015) [41]	USA	1	1	1 (M)	39	Known	Yes

**Figure 2:**
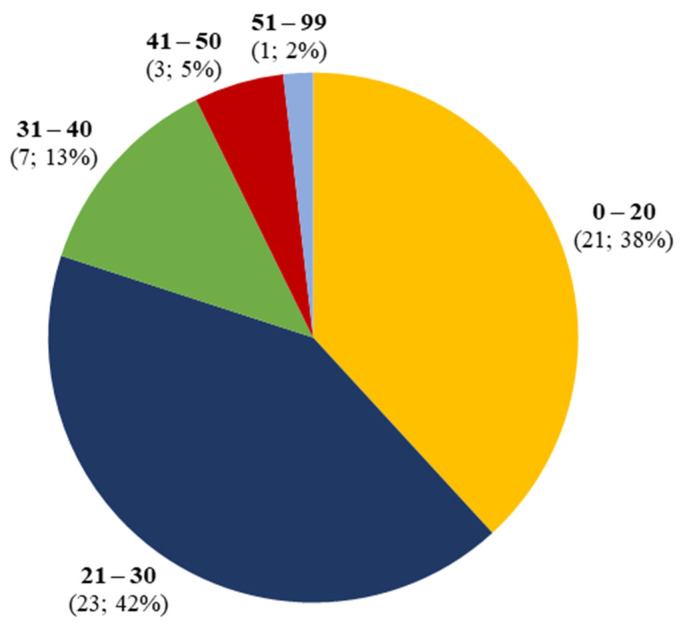
Age distribution of synthetic cannabinoid-induced acute kidney injury.

Acute kidney injury related to the use of synthetic cannabinoids was identified in 55 patients, 53 male (96%) and 2 female (4%). Mean age was 25, with a prevalence in the age group between 21 and 30 years (Figure 2).

The use of SCs was declared anamnestically in all cases; the co-intake of one or more substances including marijuana, alcohol, antipsychotic drugs, MDMA, oxycodone, and paracetamol was ascertained in eight cases.

**Figure 3:**
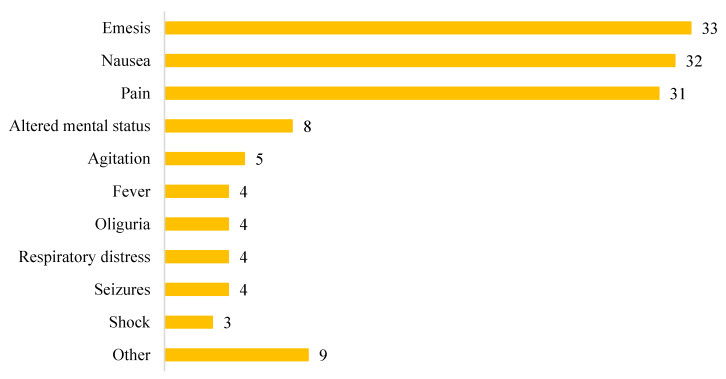
Clinical presentation of synthetic cannabinoid intoxication.

The most frequent manifestations of SC consumption (Figure 3), often jointly present, were emesis (33; 60%), nausea (32; 58%), and abdominal or flank pain (31; 56%). Neurological symptoms were found in 13 patients (24%), while urological symptoms were found in 5 (9%). Other manifestations such as headache, diarrhea, visual changes, hematuria, myalgia, paraesthesia, and anuria were found, overall, in 9% of cases.

Creatinine was measured in 49 patients (89%) as an indicator of renal function. The admission and peak creatinine values were available for 25 patients, while for 5 patients, only the admission value was reported, and for 19 patients, only the peak was inferable. The average admission creatinine value was 5.22 mg/dL, with a range from 0.66 mg/dL to 17.3 mg/dL. The mean creatinine peak was 7.62 mg/dL, with a range from 2.6 mg/dL to 21 mg/dL.

Blood urea nitrogen (BUN) was measured in 28 patients (51%). Admission and peak BUN values were available in 16 patients, while only admission values were reported in 7 patients, and only peak values were inferable in 5 patients. The average admission BUN value was 49.55 mg/dL, with a range from 9.05 mg/dL to 177 mg/dL. The mean BUN peak was 51.65 mg/dL, with a range from 23 mg/dL to 177 mg/dL.

Fractional sodium excretion (FENa) was measured as an indicator of renal function in 6 patients, with a mean of 2.73% and a range of 1.8% to 5.09%.

Serum creatinine phosphokinase (CPK) was assayed in 16 cases, with a mean peak of 51,523 U/L and a range from 255 U/L to 320,000 U/L. 

Urinalysis was performed in 23 cases (42%). Protein traces were found in 17 cases (74%), red blood cells or blood in 14 cases (61%), white blood cells in 8 cases (35%), and casts in 7 cases (30%).

Ultrasound examination was performed in 36 cases (65%). In 16 cases (44%), the examination was within normal limits; in 19 cases (53%), cortical hyperechogenicity was detected, and in 1 case (3%), there was an association of increased renal volume and cortical hyperechogenicity. CT was performed in five patients; precisely, in three cases, the examination was performed in place of the ultrasound, while in two cases, it was carried out as an additional examination.

**Figure 4:**
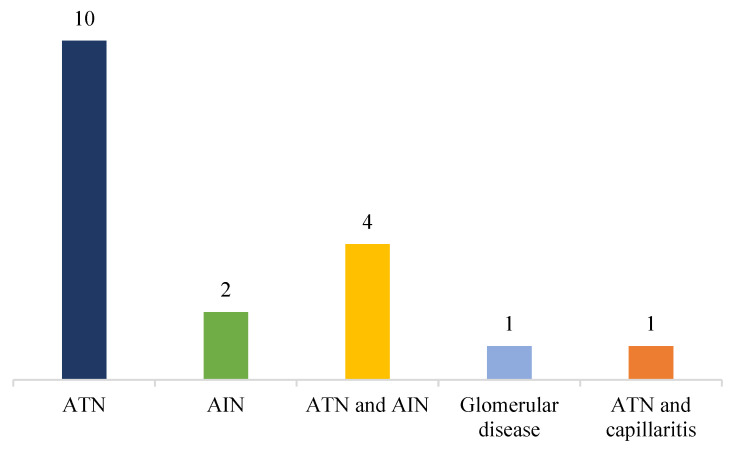
Histological findings on renal biopsy.

A renal biopsy was performed in 18 cases (33%). Histological findings were consistent with acute tubular injury in 10 cases (55%), acute tubulointerstitial nephritis in 4 cases (22%), acute interstitial nephritis in 2 cases (11%), acute tubular necrosis with peritubular medullary capillaritis in 1 case (6%), and acute glomerular disease in 1 case (6%) (Figure 4).

Time of hospitalization was available for only 17 patients (31%). Hospital stay ranged from 2 to 38 days, with a mean stay of 9 days. Hemodialysis was performed as part of therapy in 18 cases (33%).

Concerning the lethal outcome, only four patients (7%) died after SC intoxication. While, in three cases, the cause of death was attributed to a multiorgan dysfunction syndrome, in one of the cases, the cause of death was not described, as the subject arrived dead at the emergency department.

**Figure 5:**
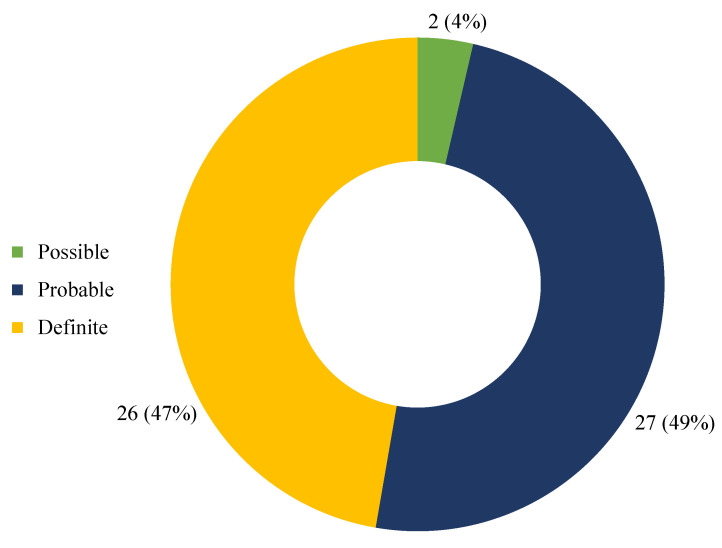
Causality assessment through Naranjo Adverse Drug Reaction Probability Scale.

The application of the Naranjo Adverse Drug Reaction Probability Scale to each case included in the study allowed the assignment of a score of 9 in 26 cases (47%), 7 in 23 cases (42%), 6 in 4 cases (7%), and 4 in 2 cases (4%); based on the obtained scores, the causal link between the intake of the substance and the adverse event was definite in 26 cases (47%), probable in 27 (49%), and possible in 2 cases (4%) (Figure 5).

## 4. Discussion

The incidence of acute kidney injury has been increasing over time, with mortality remaining high in the past decades and an estimated burden higher than that of breast cancer, heart failure, and diabetes [42,43,44]. The problem of renal function is not only related to AKI, which is normally an acute condition, but also to the tendency of people who have had AKI to develop a permanent kidney injury, which then increases the risk of developing chronic kidney disease (CKD) in the future [45]. In other words, although fraught with a low mortality rate, SC-induced AKI may result in a greater risk of CKD by integrating a significant public health problem related to long-term complications. Several scientific contributions show how the risk of developing CKD is proportional to the severity of AKI and how the latter determines an increased risk of extrarenal diseases such as hypertension and cardiovascular disease [46,47]. 

The results relating to the socio-demographic aspects of the consumption of synthetic cannabinoids are in line with the evidence relating to the consumption of cannabinoids, with a prevalence of consumption in males under the age of 30 years [48,49,50].

The geographical distribution of scientific production on the matter demonstrates a greater diagnostic suspicion of synthetic cannabinoid-induced acute kidney injury in the United States. Considering the concentration of US studies in the period 2013–2017, it is possible to correlate the increase in sensitivity towards the problem and diagnostic skills to the research, surveillance, and control activities of public health departments [51].

In the studies analyzed, the consumption of synthetic cannabinoids was established solely through anamnestic data since in none of the cases had the urinary screening tests identified the presence of metabolites. In this sense, the difficulties in detecting the urinary metabolites of synthetic cannabinoids by means of screening methods are objective and well-documented [52,53,54]. In fact, synthetic cannabinoids constitute a group of molecules—specially designed to implement the link with the receptors for endogenous cannabinoids—generally destined for a phase I oxidative enzymatic metabolism through the hepatic cytochrome P450 and, subsequently, to a phase II conjugation metabolism. A similar metabolic process determines the formation of metabolites which, also due to the continuous introduction of new substances, are scarcely predictable and are difficult to detect with urinary screening methods.

The co-intake of other substances documented in the study needs some considerations; in particular, while substances such as quetiapine and MDMA are capable of independently causing AKI, others may only facilitate its onset. In the latter case, the lack of knowledge of the interaction modalities and the continuous evolution of synthetic cannabinoids represent a challenge for understanding the pathophysiological mechanisms of acute kidney damage.

Regarding the clinical presentation, the data deriving from the systematic review are significantly consistent with the available evidence. Precisely, the association between the intake of cannabinoids and the so-called cannabinoid hyperemesis syndrome (CHS) is well-documented; the syndrome is characterized by nausea, emesis, and abdominal pain, symptoms highlighted more frequently in the cases analyzed [55,56,57,58]. Neurological symptoms were frequent, probably due to the psychotropic effects of cannabinoids [59]. In this regard, there is ample evidence that, in patients with pre-existing psychotic disorders, the use of SCs can worsen existing psychotic symptoms or facilitate the appearance of new ones [60,61,62,63]. Acute cannabinoid consumption affects cognitive integrity, inducing bizarre thinking, depersonalization [64], and psychotic symptoms [65]; similar effects result in disorders such as psychosis, depression, mania, and anxiety in prolonged consumption [66,67]. In acute intoxication, cognitive skills such as learning new information, prolonged attention, executive functions, and psychomotor integrity are also impaired [68,69]. Urological symptoms have been recorded in a limited number of cases, probably due to the fact that acute kidney damage does not present with immediate onset of overt manifestations [70].

The paucity of the nephrological and urological manifestations implies (from the diagnostic point of view) specific evaluations of renal function through the glomerular filtration rate (GFR), estimated using serum creatinine or urine production, which is more sensitive to tubular damage. According to KDIGO (Kidney Disease: Improving Global Outcomes), changes in serum creatinine levels or urine output are fundamental for the diagnosis of AKI [71]. Despite the proposals for new AKI markers, at present, the alteration of serum creatinine values still represents a cornerstone of clinical evaluation [72,73,74]. Limitations of serum creatinine dosage lie in partial insensitivity to acute changes in renal function and variability based on age, sex, muscle mass, diet, hydration status, and drugs [75,76]. Furthermore, serum creatinine is an indicator of glomerular filtration rate rather than a direct marker of tubular damage [77,78]. For these reasons, serum creatinine is considered an “imperfect gold standard” for the diagnosis of acute kidney injury [79]. The data that emerged indicate that the determination of creatinine phosphokinase (CPK) is generally performed in the suspicion of rhabdomyolysis related to acute kidney injury [80,81]. Although cases of rhabdomyolysis associated with synthetic cannabinoids have been reported in the literature, the mechanisms of muscle damage are not yet fully defined [82,83,84,85]. Typically, rhabdomyolysis is linked to the breakdown of damaged skeletal muscle; such a condition results in AKI due to intratubular obstruction from protein precipitation, inflammation, and renal vasoconstriction; therefore, the treatment of SC-induced rhabdomyolysis does not differ from the classical approach [86,87,88,89]. Solid evidence for the difficulty of identifying direct markers of kidney damage also exists for blood urea nitrogen (BUN) [90]; in fact, BUN varies with age, dietary protein content, medications, pregnancy, hydration status, congestive heart failure, gastrointestinal bleeding, shock, and severe burns. Fractional sodium excretion (FENa) is also an imperfect marker of renal function since it is influenced by daily sodium intake and glomerular filtration rate [91]; this means that the use of FENa for the evaluation of tubular damage is subject to the correction of the value for these two factors. In any case, the FENa can be useful for the classification of the AKI; in particular, pre-renal AKI cases correspond to low FENa values due to the stimulus to sodium retention related to volume depletion [92]. In the examined cases, the evaluation of FENa was always subordinated to the dosage of serum creatinine; this approach, coherently with the literature data, has contained the limits of the evaluation of the fractional excretion of sodium, allowing us to exploit its usefulness in the characterization of AKI.

The diagnostic value of renal ultrasound evaluation in patients with acute kidney injury is repeatedly described in the nephrological, radiological, and critical care literature [93,94]. The grayscale and Doppler ultrasound manifestations of acute kidney injury appear to be heterogeneous [95]. Consistent with different studies on ultrasound diagnostics, the finding most frequently highlighted in the course of AKI is an increase in cortical echogenicity [96].

The histopathology of AKI is as variable as the etiological conditions underlying the renal injury [97]. Although the incidence of AKI on renal biopsy is not fully defined, the finding of changes consistent with the diagnosis is common both in isolation and in association with other diseases. The histopathological study of the kidney biopsy is often the quickest and most effective way to trace the cause of AKI; however, it requires the involvement of expert pathologists, adequate communication with nephrologists and, sometimes, the use of specific histochemical or immunohistochemical methods [98]. Consistent with several scientific contributions, the histopathological findings most frequently identified in the studies analyzed included acute tubular necrosis and acute interstitial nephritis [99,100,101,102].

ATN and AIN probably derive from direct toxicity (increased oxidative stress at the level of tubular cells and interference with tubular transport) and indirect toxicity (indirect effects of synthetic cannabinoids) [103]. To date, the exact mechanisms of kidney damage remain unknown, also considering the unpredictable effects of other compounds mixed with the synthetic cannabinoid [104]. However, in most cases, the conceivable cause of AKI is multifactorial as well as inclusive of a pre-renal factor (excessive fluid loss related to vomiting and not replenished through adequate fluid intake), and an intrinsic factor.

In the majority of cases, hydration by intravenous infusion of fluids alone represented the effective therapeutic approach for restoring renal function [105,106]. However, constant monitoring of renal function during treatment should be considered due to the significant proportion of cases requiring hemodialysis [107]. The majority of patients resolve clinical manifestations within a few days, with an average hospital stay of 9 days noted in the present review. Despite the relatively short hospital stay, the waste of resources in emergency departments is conspicuous due to the effects of synthetic cannabinoids [108].

Regarding mortality, although the mechanisms are not yet completely understood, the consumption of synthetic cannabinoids has been associated with sudden death [109]; just consider that in some cases, despite the carrying out of an autopsy and related investigations, it is not possible to determine the pathophysiological mechanism of death. Over the years, reports of synthetic cannabinoid-related deaths have increased dramatically; in particular, deaths associated with poly-drug abuse were more frequent [110]. In partial disagreement with the literature data, the scientific contributions analyzed highlighted a relatively low number of deaths. Among other things, there is no discussion on the attribution of toxic effects to the substance or any contaminants. However, according to several reports, death related to the consumption of synthetic cannabinoids is frequently attributable to malignant ventricular arrhythmias, ischemic stroke, respiratory depression, degeneration of psychotic episodes, and reduced perception of the risk of accidents [111,112,113]; in some cases, as in the present review, death resulted from a multiorgan dysfunction syndrome with probable renal origin [114].

As regards the causal aspects of the adverse reaction to the substance, the results obtained via applying the Naranjo algorithm demonstrated a quantitatively significant correlation between the intake of synthetic cannabinoids and the onset of acute kidney injury. In particular, in the majority of cases, the adverse reaction showed a reasonable temporal relationship with the intake of the synthetic cannabinoid, regressed after discontinuation, and consisted of a clinically appreciable deterioration in renal function. Frequently, the precise toxicological determination of the substance has been difficult due to an anamnestic defect, having generally been reported as the generic intake of a synthetic cannabinoid. This issue is extremely important in improving diagnostic procedures and patient safety in consideration of the heterogeneity and evolution of the category of synthetic cannabinoids.

The synthesis of the evidence conducted in the present study permitted us to focus attention on a further aspect of synthetic cannabinoid-induced organic damage. Specifically, a large number of studies address cannabinoid-induced liver damage [115]. As well as for kidney damage, an increasing number of studies in recent years are addressing the problems of drug-induced liver injury (DILI) and herbal-induced liver damage (HILI), highlighting, as already mentioned, growing interest from health professionals [116,117]. DILI represents a condition of significant health importance involving professionals from different disciplines. The condition has an annual incidence in the general population of 1–2 cases per 100,000 individuals [118]; the highest incidence worldwide is described in China with 23.8 per 100,000 subjects. On the contrary, HILI represents a condition that is challenging to interpret due to the presence in herbal products of numerous ingredients, the often-concomitant intake of different products, and mislabeling [119]. According to different studies, the condition’s incidence varies between 1.16 per 100,000 in the United States and 6.38 per 100,000 in mainland China [120,121]. In addition to the pathophysiological and diagnostic aspects, the evaluation of causality plays a very important role in the interpretation of DILI and HILI, as demonstrated by the application of the Naranjo algorithm to cannabinoid-induced kidney damage. For this reason, the Roussel Uclaf Causality Assessment Method (RUCAM) [122] was designed in 1993 and is still today the most widely used causality assessment tool in DILI and HILI. Precisely, if RUCAM facilitates the diagnosis of DILI on one hand, on the other hand, it is extremely useful in reducing uncertainties in the evaluation of HILI. Over the years, the use of RUCAM has produced significant results by giving objectivity to the diagnosis of DILI, which is notoriously based on a criterion of exclusion of other causes [123]; likewise, the prospects are encouraging regarding the application of RUCAM to HILI. Considering the contribution of the causality evaluation in the interpretation of substance-induced liver and kidney damage, it seems desirable to continue and implement research activity aimed at consolidating the evidence currently available [124,125]. Similarly, given the considerable clinical interest and the repercussions in terms of public health related to the constant evolution and growing diffusion of synthetic cannabinoids, it seems appropriate to carry out further studies on the possible pathophysiological interactions between liver and kidney in the genesis and manifestations of damage.

Conclusively, the diagnosis of acute kidney injury related to synthetic cannabinoids currently represents a challenge both in the clinical and forensic fields. Diagnosis is frequently challenging due to the different clinical presentations and the continual introduction of new substances [126]. Synthetic cannabinoid-related acute kidney injury should be suspected, in the absence of other causes, in all subjects with elevated serum creatinine, even when drug screening tests are negative. Prompt identification and treatment of the condition represent a sensitive public health goal both for the acute management of damage from synthetic cannabinoids and for the prevention of chronic kidney disease [127,128].

## Data Availability

The data used in the present study are available from the corresponding author upon request.
